# Fractionating the anterior temporal lobe: MVPA reveals differential responses to input and conceptual modality

**DOI:** 10.1016/j.neuroimage.2016.11.067

**Published:** 2017-02-15

**Authors:** Charlotte Murphy, Shirley-Ann Rueschemeyer, David Watson, Theodoros Karapanagiotidis, Jonathan Smallwood, Elizabeth Jefferies

**Affiliations:** Department of Psychology and York Neuroimaging Centre, University of York, UK

**Keywords:** Anterior temporal lobe (ATL), Multi voxel pattern analysis (MVPA), Semantic, Hub, Spoke, Resting-state connectivity

## Abstract

Words activate cortical regions in accordance with their modality of presentation (i.e., written vs. spoken), yet there is a long-standing debate about whether patterns of activity in any specific brain region capture modality-invariant conceptual information. Deficits in patients with semantic dementia highlight the anterior temporal lobe (ATL) as an amodal store of semantic knowledge but these studies do not permit precise localisation of this function. The current investigation used multiple imaging methods in healthy participants to examine functional dissociations within ATL. Multi-voxel pattern analysis identified spatially segregated regions: a response to input modality in anterior superior temporal gyrus (aSTG) and a response to meaning in more ventral anterior temporal lobe (vATL). This functional dissociation was supported by resting-state connectivity that found greater coupling for aSTG with primary auditory cortex and vATL with the default mode network. A meta-analytic decoding of these connectivity patterns implicated aSTG in processes closely tied to auditory processing (such as phonology and language) and vATL in meaning-based tasks (such as comprehension or social cognition). Thus we provide converging evidence for the segregation of meaning and input modality in the ATL.

## Highlights

• Multi-voxel pattern analysis identified spatially segregated regions in the anterior temporal lobe.
• Anterior superior temporal gyrus (aSTG) responded to modality input.
• Ventral anterior temporal lobe (vATL) responded to semantic meaning.
• Converging findings from resting-state connectivity supports this functional dissociation.

## 1 Introduction

Current neurocognitive models propose that concepts are represented in a large-scale distributed network comprising (1) sensory and motor ‘spoke’ regions that store knowledge of physical features and (2) convergence zones that integrate across multiple modalities (e.g., visual vs. auditory) to form abstract amodal representations (Damasio, 1989, Patterson et al., 2007). For example, the hub and spoke model of Patterson et al. (2007) proposes that information from modality-specific spoke regions is integrated in an amodal ‘hub’ region within the anterior temporal lobes (ATL), allowing the conceptual similarity of items that are semantically similar yet share few surface features, such as ‘flute’ and ‘violin’, to be represented, and making it possible to map between modalities so that we can picture a flute and imagine the sound that it makes from only its name (e.g., Damasio, 1989; Lambon et al., 2010; Patterson et al., 2007; Rogers et al., 2004). This hub and spoke model proposes that both the ATL and modality-specific spokes make a crucial contribution to conceptual representation, and these elements are mutually-constraining through a pattern of interactive-activation.

The spokes are hypothesized to represent the contributions of sensory and motor cortex to conceptual knowledge, as words associated with specific sensorimotor attributes activate corresponding sensorimotor cortex. For example, words denoting actions (e.g., kick) activate the motor system (Postle et al., 2008, Rueschemeyer et al., 2007, Rueschemeyer et al., 2010), while words associated with specific smells (e.g., cinnamon) elicit activation in olfactory cortex (Cerf-ducastel and Murphy, 2004, Gonzalez et al., 2006). Although these neural regions are important for perception and action, they are also recruited during semantic processing to provide meaning to words (Barsalou, 1999, Barsalou, 2008, Martin, 2007, Patterson et al., 2007, Kiefer and Pulvermuller, 2012).

The proposal that the ATL forms a key semantic “hub” capturing knowledge across different input modalities was initially put forward to account for the pattern of impairment in semantic dementia (SD), in which relatively focal atrophy centered on ATL leads to progressive conceptual degradation across modalities and tasks (e.g., Patterson et al., 2007; Rogers et al., 2015). SD patients are highly consistent in the knowledge they can demonstrate when the same concepts are probed in different ways, suggesting central semantic representations degrade in this condition. Patients with SD have atrophy which increasingly affects inferior frontal and posterior temporal areas, as well as ATL, making it difficult to draw strong conclusions about the location of the “hub” from neuropsychology alone; however, the severity of the semantic impairment correlates most strongly with the degree of hypometabolism in inferior ATL (Mion et al. 2010). The crucial role of ATL is also supported by functional neuroimaging studies of healthy participants that show amodal conceptual processing in ATL (Rice et al., 2015; Visser et al., 2010). For example, Visser and Ralph (2011) characterized the degree of modality convergence in STG, MTG, ITG and fusiform cortex comparing posterior and anterior parts of the temporal lobe. Both STG and fusiform were modality-sensitive along the temporal lobe, showing stronger activation for spoken words and pictures respectively. MTG showed a multimodal response in both anterior and posterior regions. ITG uniquely showed a pattern consistent with the increasing integration of information from different inputs, namely sensitivity to modality in posterior but not anterior regions. Moreover, Spitsyna et al. (2006) showed that, despite originating from different sensory inputs, there is considerable activation overlap for spoken and written processing in ATL regions. Thus, emerging evidence from both patients with SD and healthy participants suggests that the semantic hub may be located in ventral ATL.

These observations raise the possibility of functional dissociations with ATL. Jackson et al. (2016) recently observed different patterns of functional connectivity within superior and ventral regions of the ATL, with anterior STG showing stronger connectivity to language, auditory and motor regions, while ventral ATL showed connectivity to other multimodal semantic regions including inferior frontal gyrus, angular gyrus and posterior middle temporal gyrus. These parallel the pattern of white-matter connections found by Binney et al., (2012) and Jung et al., (2016). Consistent with these findings it has been proposed that superior regions of the ATL are important in lexical and auditory processing, while ventral regions support conceptual processing across all sensory modalities (Rice et al., 2015; Visser et al., 2010; Visser and Ralph, 2011). Ventral and ventrolateral ATL regions have been found to respond to meaningful inputs across multiple modalities by studies employing convergent methods; including fMRI and transcranial magnetic stimulation (Binney et al., 2010, Visser et al., 2012, Hoffman et al., 2015) and representational similarity analysis (RSA) of ECoG data (Chen et al., 2016).

The current study used multiple imaging methodologies to simultaneously investigate the organization of knowledge in the ATL (hub) and auditory and visual regions (as potential spokes). In a functional experiment we manipulated the format in which words were presented (i.e., spoken, written) and the modality-specific features associated with the word's meaning (e.g., auditory features: “loud” vs. visual features: “shiny”). We used Multi Voxel Pattern Analysis (MVPA) to decode how these different features (modality of presentation and underlying meaning) are represented. Based on the hub and spoke model, we expected this analysis to reveal regions that are distributed across the cortex that responded to the meaning of the stimulus regardless of the input modality. In this experiment we were particularly interested in identifying regions in ATL where the meaning of words is represented that are independent of input modality. The amodal hub regions should be able to code the meaning of a stimulus regardless of the presentation format (e.g., auditory feature words should elicit similar patterns of activation even when spoken and written words are compared). In addition, this region should represent the meaning of words tied to different sensory modalities (i.e., it should represent words with auditory meanings like ‘loud’ and words with visual meanings like ‘shiny’). In contrast, the spokes should represent particular semantic features in regions of sensory cortex (i.e., words with an auditory meaning, such as loud, should be represented in auditory cortex regardless of how they are presented (written or spoken). However, spoke regions are not expected to represent meaning that is tied to a different sensory modality (i.e., auditory cortex may not contribute to semantic representation for words with a visual meaning, such as shiny).

Next we used the regions identified in our MVPA analysis as regions of interest in a seed based resting state connectivity analysis to understand the neural networks in which these different regions of the ATL are embedded. We expected the amodal region of ATL to show functional connectivity with regions of cortex that are important in more abstract forms of cognition, e.g., the default mode network, rather than regions important in unimodal sensory processing, such as the auditory and visual cortex. Finally, we used the search tool Neurosynth to decode the most common interpretations of this pattern of functional connectivity in the broader neuroimaging literature.

## 2 Materials and methods

### 2.1 Functional experiment

#### 2.1.1 Participants

Twenty participants were recruited from the University of York. One participant's data was excluded due to excessive motion artifacts, leaving nineteen subjects in the final analysis (10 female; mean age 24.55, range 18–36 years). Participants were native British speakers, right handed and had normal or corrected-to-normal vision. Participants gave written informed consent to take part and were reimbursed for their time. The study was approved by the York Neuroimaging Centre Ethics Committee at the University of York.

#### 2.1.2 Stimuli

**Fig. 1:**
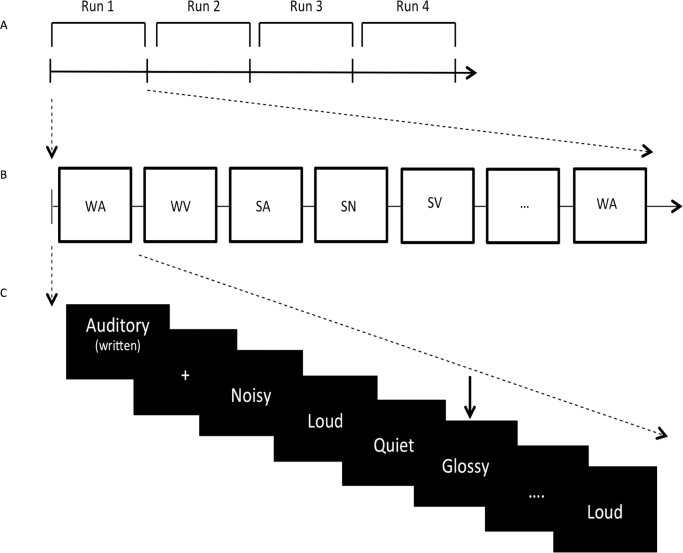
Experimental design. (A) Four runs across the fMRI session. Each run lasted no longer than 6 min 19 s. (B) Block organization across each run. WA=written-Aud, WV=written-VIS, WN=written-NON, SA=spoken-AUD, SV=spoken-VIS and SN=spoken-NON. Only 6 are depicted for illustration (from a total of 12 blocks). Each of the 6 conditions were randomly presented twice, with no immediate repetition. Written blocks lasted 22.7 seconds, spoken blocks lasted no longer than 23.2 seconds. (C) Each block began with written instructions stating the semantic feature type and presentation format, for 3500ms (followed by 500 ms fixation). The 8 items from the condition were then presented twice in a random order, with no immediate repetition. Only 5 are depicted for illustration (from a total of 16 items). The arrow represents an out-of-category item (e.g., visual feature ‘glossy’ in a block of auditory features). In total, 17 words were presented within each block (16 targets and 1 catch trial).

Participants were presented with blocks of spoken and written items from three conditions: AUD words denoted auditory features (e.g., loud), VIS words denoted visual features (e.g., shiny) and NON stimuli were meaningless nonwords (e.g., brodic). A block consisted of a sequence of items; participants were asked to pay attention to the meaning of each item, and respond with their left index finger when an out-of-category item was presented (see Fig. 1). For VIS and AUD blocks, half of the out-of-category items were taken from the non-presented feature condition, while the other half were taken from a separate list of taste words (e.g., spicy). Participants could not predict the category of the out-of-category item and therefore had to focus on the AUD or VIS feature specified in the instructions. In the NON condition, participants were asked to respond to any item that was a word. All stimuli were presented in both spoken and a written format. Spoken words were recorded digitally and then normalized for volume and power. Written words were presented centrally as white letters on a black background. The combination of item meaning (AUD, VIS, NON) and presentation format (Spoken, Written) yielded 6 experimental conditions (Spoken-AUD, Spoken-VIS, Spoken-NON, Written-AUD, Written-VIS, Written-NON).

The selection of AUD and VIS words was validated in a behavioural study with twelve participants who did not take part in the fMRI session. Participants were asked to rate a subset of modality-specific words (n=220), according to how much each one related to four sensory categories; auditory, visual, haptic and taste. Participants also provided ratings of familiarity and emotional valence. All ratings were given on a 5-point likert-scale. We selected adjectives with strong auditory or visual associations. Each set contained 8 items which were matched for key psycholingusitic variables such as frequency and length (see Table 1; Wilcoxon signed rank tests revealed all *p*>.05). AUD words (such as ‘loud’) were selected if they scored significantly higher on the auditory than visual, haptic or taste modalities (all p<.001). Likewise VIS words (such as ‘shiny’) were selected if they scored significantly higher on the visual than the auditory, haptic or taste modalities (all *p*<.001).

A set of 8 taste features were used in out-of-category catch trials. These items scored significantly higher on the taste modality than auditory, visual and haptic (p<.001). These items were also matched to AUD or VIS words on the variables in Table 1 (all *p*>.05). Finally, NON words were made by recombining the phonemes from the AUD and VIS conditions to create 8 pseudo-words. The non-word condition matched AUD and VIS conditions on number of letters, syllable and Levenshtein distance (Levenshtein, 1965), which quantifies the number of phoneme insertions, deletions and/or substitutions required to change one word into another, (all p>.05). The use of a small number of items is consistent with other MVPA studies into semantic representation (Correia et al., 2014; Peelen and Caramazza, 2012).

Stimulus presentation was controlled by a PC running Neurobehavioural System Presentation® software (Version 0.07, www.neurobs.com). Stimuli were projected onto a screen viewed though a mirror mounted on the head coil. Spoken stimuli were presented binaurally using MR-compatible headphones.

#### 2.1.3 Task procedure

Prior to being scanned, participants were shown a printed copy of all stimuli (8 AUD, 8 VIS, 8 NON) to familiarize them with the items. They also performed a practice session consisting of 12 blocks, identical to one scanning run.

In the scanner there were 4 runs of 12 blocks. The choice of 4 functional runs is consistent with many MVPA studies that also presented trials within 4 runs that were each 5–10 min long (Coutanche and Thompson-Schill, 2012; Fairhall and Caramazza, 2013; Peelen and Caramzza, 2012). Within each run, there were two blocks related to each of the 6 experimental conditions (spoken and written words combined with three meaning conditions: AUD, VIS and NON). These were presented in a pseudo-random order, with no immediate repetition of conditions. Blocks were separated by a jittered gap (4–8 s) during which a red fixation cross was presented. A block consisted of 17 stimuli: eight stimuli related to that experimental condition presented twice in a pseudo-random order, with no immediate repetition, plus one out-of-category catch trial. Written stimuli were presented for 600ms; spoken stimuli were presented on average for 633.57 ms (SD=71.57 ms). Words within each block were separated by a 500 ms inter-stimulus interval.

Block transitions were marked with a written task instruction, which indicated (i) the aspect of meaning that participants needed to focus on and (ii) the presentation format presented in parentheses. The task instructions were presented for 3500 ms (followed by 500ms fixation). A grey fixation cross against a black background was used to minimize eye movements during the duration of a block. Each block (including task instruction and jittered rest period) lasted on average 28.7 s.

#### 2.1.4 Acquisition

Data were acquired using a GE 3 T HD Excite MRI scanner at the York Neuroimaging Centre, University of York. A Magnex head-dedicated gradient insert coil was used in conjunction with a birdcage, radio-frequency coil tuned to 127.4 MHz. A gradient-echo EPI sequence was used to collect data from 38 bottom-up axial slices aligned with the temporal lobe (TR=2 s, TE=18 ms, FOV=192×192 mm, matrix size=64×64, slice thickness=3 mm, slice-gap 1mm, flip-angle=90°). Voxel size was 3×3×3 mm. Functional images were co-registered onto a T1-weighted anatomical image from each participant (TR=7.8 s, TE=3 ms, FOV=290 mmx290 mm, matrix size=256 mmx256 mm, voxel size=1.13 mmx1.13 mmx1 mm) using linear registration (FLIRT, FSL).

#### 2.1.5 Preprocessing

Imaging data were preprocessed using the FSL toolbox (http://www.fmrib.ox.ac.uk/fsl). Images were skull-stripped using a brain extraction tool (BET, Smith, 2002) to remove non-brain tissue from the image. The first five volumes (10 s) of each scan were removed to minimize the effects of magnetic saturation, and slice-timing correction was applied. Motion correction (MCFLIRT, Jenkinson et al., 2002) was followed by temporal high-pass filtering (cutoff=0.01 Hz). Individual participant data were first registered to their high-resolution T1-anatomical image, and then into a standard space (Montreal Neurological Institute (MNI152); this process included tri-linear interpolation of voxel sizes to 2×2×2 mm. For univariate analyses, data were additionally smoothed (Gaussian full width half maximum 6 mm).

#### 2.1.6 Univariate analysis

The condition onset and duration were taken from the first item in each block (after the initial instructions) to the end of the last item. The response to each of the 6 conditions was contrasted against rest. Box-car regressors for each condition, for each run, in the general linear model were convolved with a double gamma hemodynamic response function (FEAT, FSL). Regressors of no interest were also included to account for head motion within scans. A fixed effect design (FLAME, http://www.fmrib.ox.ac.uk/fsl) was then conducted to average across the four runs, within each individual. Finally, individual participant data were entered into a higher-level group analysis using a mixed effects design (FLAME, http://www.fmrib.ox.ac.uk/fsl) whole-brain analysis.

#### 2.1.7 Multivariate pattern analysis

Parameter estimates were calculated in the same manner as for univariate analyses, for each condition and for each run: in this way, the spatial pattern information entered into the classifier from each condition represented the average response to the 8 exemplars. This method is consistent with previous literature investigating semantic representations (Coutanche & Thompson-Schill, 2012; Fairhall and Caramazza, 2013; Peelen and Caramagzza, 2012): it allows us to make inferences that a particular region is able to discriminate between words referring to auditory and visual features, for example, but not the meanings of these individual words. MVPA was conducted on spatially unsmoothed data to preserve local voxel information.

As we had a priori knowledge of strong selectivity for the classes in particular brain regions (ATL, primary auditory cortex and primary visual cortex), we opted for a ROI-based MVPA method rather than whole-brain analysis. This reduced the number of voxels used for classification (and therefore the number of free parameters which can lead to over-fitting; for similar approaches see Kamitani and Tong (2005) and Kuhl, Rissman, Chun and Wagner, (2011). The following masks were used; primary visual cortex (taken from FSL Juelich Atlas; http://fsl.fmrib.ox.ac.uk/fsl/fslwiki/Atlases), primary auditory cortex (taken from FSL Juelich Atlas; http://fsl.fmrib.ox.ac.uk/fsl/fslwiki/Atlases) and ATL (anterior to Y=−22; Hoffman et al., 2015). The size of these masks are as follows; primary visual cortex, 12662 voxels; primary auditory cortex, 2372 voxels; ATL, 18523 voxels.

To ensure that our ROIs had sufficient signal to detect reliable fMRI activation, the temporal signal-to-noise ratio (tSNR) for each participant was calculated for the first run of the experiment by dividing the mean signal in each voxel by the standard deviation of the residual error time series in that voxel (Friedman et al., 2006). tSNR values were averaged across the voxels of each ROI. Mean tSNR values, averaged across participants, were as follows: ATL, 76.74; primary auditory cortex (PAC), 93.61; primary visual cortex (PVC), 102.96. The percentage of voxels in each ROI that had “good” tSNR values (>20; Binder et al., 2011) was above 85% for all ROIs: ATL, 86.17%; PAC, 99.87%; PVC, 94.58%. These values indicate that, although mean tSNR was lower in anterior temporal cortex than in sensory regions, the tSNR was sufficient to detect reliable fMRI activation in all ROIs (Binder et al., 2011). Moreover, to determine whether tSNR was sufficient in each sub-region of the ATL (as signal drop out is most prominent in ventral anterior regions), the tSNR was calculated for the following regions: aSTG, 85.97; aMTG, 89.00; aITG, 69.79; anterior fusiform gyrus, 69.74; anterior parahippocampal gyrus, 67.13; temporal pole, 63.27. These values suggest that, again, although mean tSNR was lower in more ventral anterior regions, it was still sufficient to detect reliable fMRI activation (Binder et al., 2011).

For each voxel in our three ROI masks, we computed a linear support vector machine (LIBSVM; with fixed regularization hyper-parameter C=1) and a 4-fold cross-validation (leave-one-run-out) classification, implemented in custom python scripts using the pyMVPA software package (Hanke et al., 2009). A support vector machine was chosen as this aims to combat over-fitting by limiting the complexity of the classifier (Lewis-Peacock and Norman, 2013). The classifier was trained on three runs and tested on the independent fourth run; the testing set was then alternated for each of four iterations. Classifiers were trained and tested on individual subject data transformed into MNI standard space. The functional data were first z-scored per voxel within each run. The searchlight analysis was implemented by extracting the z-scored β-values from spheres (6mm radius) centered on each voxel in the masks. This sized sphere included∼1233 mm voxels (Kriegeskorte et al., 2006). Classification accuracy (proportion of correctly classified trials) for each sphere was assigned to the sphere's central voxel, in order to produce accuracy maps. The resulting accuracy maps were then smoothed with a Gaussian kernel (6mm FWHM). To determine whether accuracy maps were above chance-levels (50%), individual accuracy maps were entered into a higher-level group analysis (mixed effects, FLAME; http://www.fmrib.ox.ac.uk/fsl), testing the accuracy values across subjects against chance for each voxel. Voxel inclusion was set at z=2.3 with a cluster significance threshold at FWE *p*<.05.

**Fig. 2:**
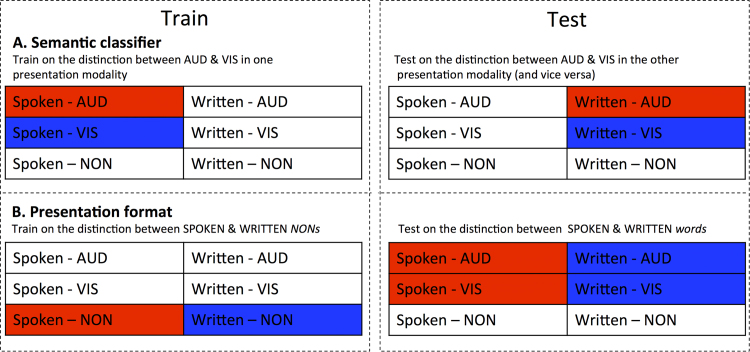
Schematic illustration of the MVPA searchlight classifiers performed. Each box includes the six experimental conditions. Classifiers were trained to distinguish between two conditions (red and blue). The classifiers were then tested on independent trials that differed in the same way. (A) Classifiers were trained and tested based on semantic content (trained on Spoken-AUD vs. Spoken-VIS, tested on Written-AUD vs. Written-VIS – and vice versa). The results from both comparisons were then averaged. (B) Classifiers were trained and tested based on presentation format (trained on Spoken-NON vs. Written-NON, tested on Spoken words vs. Written words). (For interpretation of the references to color in this figure legend, the reader is referred to the web version of this article).

The following classification tests were performed: (1) *Semantic feature classifier*: this examined whether patterns of activity conveyed information regarding the meanings of words, by training a classifier to discriminate between words referring to auditory features (e.g. loud) and visual features (e.g., shiny). This classifier was truly format-independent in the sense that it was trained on this semantic distinction using spoken words and tested using written words (and vice versa). The advantage of performing the classification in this manner is only semantic information common to both presentation formats was informative to the classifier (see Fig. 2A). The results from the two classifications were averaged to produce a single estimate of classification accuracy. (2) *Perceptual classifier*: here a classifier was trained to discriminate between spoken and written non-words and was tested on these two presentation formats for words. In this way only the presentation format that was general to both non-words and words was informative to the classifier (see Fig. 2B).

### 2.2 Resting state fMRI

#### 2.2.1 Participants

This analysis was performed ona separate cohort of 42 healthy participants at York Neuroimaging Centre (13 male; mean age 20.31, range 18–25 years). Subjects completed a 9 minute functional connectivity MRI scan during which they were asked to rest in the scanner with their eyes open. Using these data we examined the resting-state fMRI (rs-fMRI) connectivity of ATL regions that were informative to the semantic feature (aITG) and perceptual classifiers (aSTG) to investigate whether these regions fell within similar or distinct networks. In addition, we investigated the rs-fMRI connectivity of semantic regions within primary sensory cortices that showed significant decoding by the semantic classifiers to examine whether these regions overlap with the connectivity maps of the ATL seeds.

#### 2.2.2 Acquisition

As with the functional experiment, a Magnex head-dedicated gradient insert coil was used in conjunction with a birdcage, radio-frequency coil tuned to 127.4 MHz. For the resting-state data, a gradient-echo EPI sequence was used to collect data from 60 axial slices with an interleaved (bottom-up) acquisition order with the following parameters: TR=3 s, TE=minimum full, volumes=180, flip angle=90°, matrix size=64×64, FOV=192×192 mm, voxel size=3x3×3 mm. A minimum full TE was selected to optimise image quality (as opposed to selecting a value less than minimum full which, for instance, would be beneficial for obtaining more slices per TR). Functional images were co-registered onto a T1-weighted anatomical image from each participant (TR=7.8 s, TE=3 ms, FOV=290 mmx290 mm, matrix size=256 mm x256 mm, voxel size=1 mmx1 mmx1 mm).

#### 2.2.3 Pre-processing

Data were preprocessed using the FSL toolbox (http://www.fmrib.ox.ac.uk/fsl). Prior to conducting the functional connectivity analysis, the following pre-statistics processing was applied to the resting state data; motion correction using MCFLIRT to safeguard against motion-related spurious correlations (Baker et al., 2015, Smallwood et al., 2016, Krieger-Redwood et al., 2016, Davey et al., 2016); slice-timing correction using Fourier-space time-series phase-shifting; non-brain removal using BET; spatial smoothing using a Gaussian kernel of FWHM 6 mm; grand-mean intensity normalisation of the entire 4D dataset by a single multiplicative factor; high-passtemporalfiltering (Gaussian-weighted least-squares straight line fitting, with sigma=100 s); Gaussian lowpass temporal filtering, with sigma=2.8 s.

#### 2.2.4 Low level analysis

For our ATL sites we created two spherical seed ROIs, 6 mm in diameter,centered on the co-ordinates of the central voxel in the highest performing spheres in our presentation and semantic searchlight analyses; left aSTG [-54 2 -10] and aITG [-50 -10 -26] respectively (see Table 2). For our sensory semantic regions we created two spherical seed ROIS centered on intracalcarine cortex [-18 -84 4] and planum polare [-48 -12 -4] from the best performing spheres in our semantic searchlight analysis; as these regions showed high performance accuracy on the semantic classifier *and* fall within primary sensory regions.

The time series of these regions were extracted and used as explanatory variables in a separate subject level functional connectivity analysis for each seed. Subject specific nuisance regressors were determined using a component based noise correction (CompCor) approach (Behzadi et al., 2007). This method applies principal component analysis (PCA) to the fMRI signal from subject specific white matter and CSF ROIS. In total there were 11 nuisance regressors, five regressors from the CompCorr and a further 6 nuisance regressors were identified using the motion correction MCFLIRT. These principle components are then removed from the fMRI data through linear regression. The WM and CSF covariates were generated by segmenting each individual's high-resolution structural image (using FAST in FSL; Zhang et al., 2001). The default tissue probability maps, referred to as Prior Probability Maps (PPM), were registered to each individual's high-resolution structural image (T1 space) and the overlap between these PPM and the corresponding CSF and WM maps was identified. These maps were then thresholded (40% for the SCF and 66% for the WM), binarized and combined. The six motion parameters were calculated in the motion-correction step during pre-processing. Movement in each of the three Cartesian directions (x, y, z) and rotational movement around three axes (pitch, yaw, roll) were included for each individual.

#### 2.2.5 High level analysis

At the group-level the data were processed using FEAT version 5.98 part of FSL (FMRIB's Software Library,www.fmrib.ox.ac.uk/fsl) and the analyses were carried out using FMRIB's Local Analysis of Mixed Effects (FLAME) stage 1 with automatic outlier detection. The z statistic images were then thresholded using clusters determined by z > 2.3 and a (corrected) cluster significance threshold of *p* = 0.05 (Worsley, 2001). No global signal regression was performed.

To investigate the differences between the connectivity maps a fixed effect design (FLAME, http://www.fmrib.ox.ac.uk/fsl) was conducted for each participant to investigate four contrasts; (i) aSTG>aITG seed, (ii) aITG>aSTG seed, (iii) auditory semantic>visual semantic seed and (iv) visual semantic>auditory semantic seed. Individual participant data were then entered into a higher-level group analysis using a mixed effects design (FLAME, http://www.fmrib.ox.ac.uk/fsl) whole-brain analysis. Finally, to determine whether our ATL seeds connectivity maps overlap with the connectivity maps of the sensory semantic seeds we calculated the number of overlapping voxels for our two ATL sites and the sensory semantic connectivity maps.

### 2.3 Resting state decoder

To allow quantitative inferences to be drawn on the functional neural activity identified through our seed based correlational analyses we performed an automated meta-analysis using NeuroSynth (http://neurosynth.org/decode; Yarkoni et al., 2011). This software computed the spatial correlation between each ATL component mask and every other meta-analytic map (*n*=11406) for each term/concept stored in the database (e.g., semantic, language, memory, sensory). The 15 meta-analytic maps exhibiting the highest positive correlation and negative correlation for each sub-system mask were extracted, and the term corresponding to each of these meta-analyses is shown in Fig. 4. The font size reflects the size of the correlation (ranging from *r*=0.10 to 0.45 for positive correlations and *r*=−0.05 to −0.2 for negative correlations, in increments of 0.05). This allows us to quantify the most likely reverse inferences that would be drawn from these functional maps by the larger neuroimaging community.

## 3 Results

### 3.1 Behavioural results

Accuracy and reaction times (RT) were calculated for each participant (n=19) for the catch trials in each experimental condition. Results showed that all participants paid attention to the words as indicated by a mean accuracy above 80% for all experimental conditions (spoken AUD = 80.63% ± 15.33, spoken VIS = 88.12% ± 4.86, spoken NON=85.62%±11.47, written AUD=83.12%±19.01, written VIS=86.25%±13.52, written NON=88.75%±5.45). A chi-square test of independence revealed that accuracy did not significantly differ across the six experimental conditions ($x^{2}$(5)=6.09, p=.303) or across spoken and written input ($x^{2}$(1)=.301, ns). RTs differed significantly between modality-input (*t*(59)=7.36, p<.001), but not semantic-category within each modality (spoken: F(2,38)=.92, ns; written: F(2,38)=0.074, ns). In line with previous findings (Booth et al., 2002, Cohen et al., 2004), participants were significantly faster at responding to written than spoken stimuli. Furthermore, there was no difference in RT between AUD, VIS and NON items within each presentation modality, suggesting that the experimental conditions were well matched at the behavioural level within our stimuli subset.

### 3.2 Searchlight analysis

#### 3.2.1 Semantic feature classifier

**Fig. 3:**
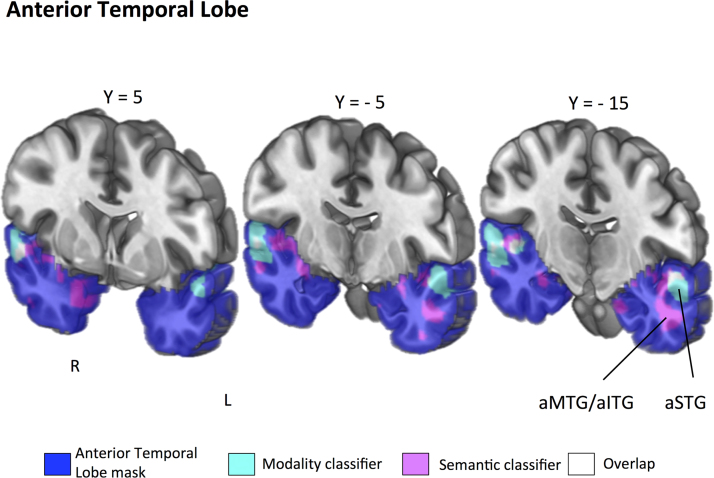
Coronal slices taken at Y=5, Y=−5 and Y=−15. Anterior temporal lobe mask shows all regions of the temporal lobe anterior to Y=−22 in line with Hoffman et al., (2015) projected in blue. Results of the group-level searchlight analysis for *semantic feature classification* (AUD vs. VIS) projected in magenta (cluster-corrected *p*<.01). Results for *perceptual classifier* (spoken vs. written) projected in cyan (cluster-corrected *p*<.01). Overlap of the two searchlight analyses in white. In total 47 voxels overlapped across the two searchlight analyses in aSTG (right hemisphere, 38 voxels; left hemisphere, 9 voxels). aSTG=anterior superior temporal gyrus; aMTG/aITG=anterior middle temporal gyrus/inferior temporal gyrus. (For interpretation of the references to color in this figure legend, the reader is referred to the web version of this article.).

**Fig. 4:**
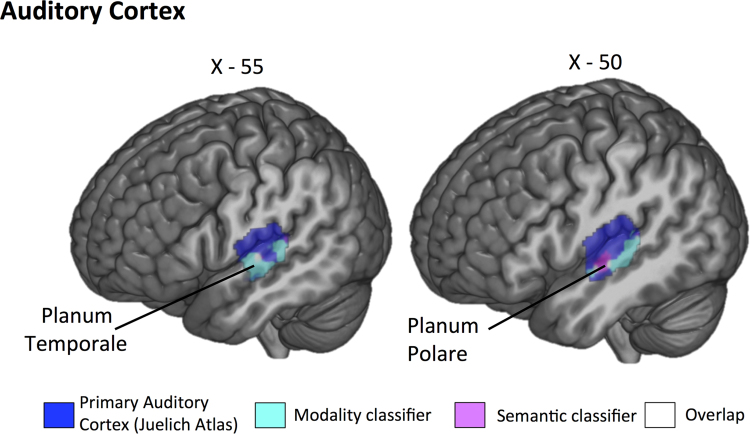
Left hemisphere sagittal slices taken at X=−55 and X=−50. Primary auditory ROI taken from Juelich histological atlases projected in blue. Results of the group-level searchlight analysis for *semantic feature classification* (AUD vs. VIS) projected in magenta (cluster-corrected *p*<.01). Results for *perceptual classifier* (spoken vs. written) projected in cyan (cluster-corrected *p*<.01). Overlap of the two searchlight analyses in white. (For interpretation of the references to color in this figure legend, the reader is referred to the web version of this article.).

**Fig. 5:**
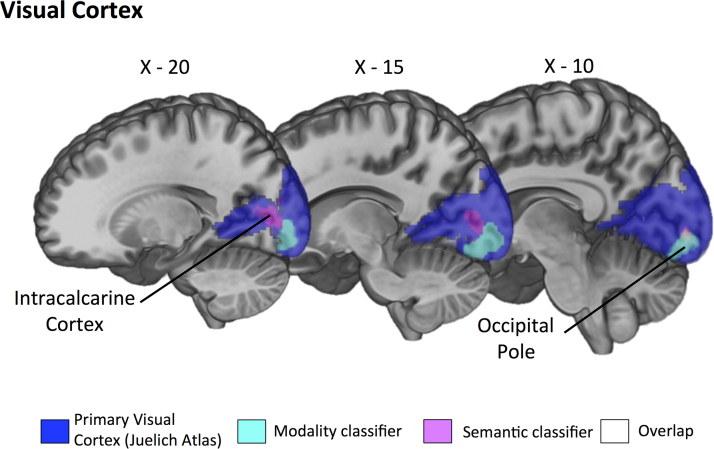
Left hemisphere sagittal slices taken at X=−20, X=−15 and X=−10. Primary visual ROI taken from Juelich histological atlases projected in blue. Results of the group-level searchlight analysis for *semantic feature classification* (AUD vs. VIS) projected in magenta (cluster-corrected *p*<.01). Results for *perceptual classifier* (spoken vs. written) projected in cyan (cluster-corrected *p* < .01). Overlap of the two searchlight analyses in white. (For interpretation of the references to color in this figure legend, the reader is referred to the web version of this article.).

The format-independent searchlight classifier, trained on the distinction between visual and auditory features in one presentation modality and tested on this distinction in the other modality, was run in three separate masks (ATL; primary auditory cortex and primary visual cortex). All results reported are above chance levels (50%, cluster corrected *p*<.05). The searchlight analysis within the ATL mask revealed a left hemisphere cluster that could decode semantic information across modalities in aMTG and aITG (see Fig. 3, Table 2). Additionally, right hemisphere clusters were revealed in anterior parahippocampal gyrus and temporal pole (TP). The searchlight analysis within the primary auditory mask revealed a cluster in planum polare (see Fig. 4, Table 2). Finally, the primary visual cortex mask revealed a cluster in intracalcarine cortex that could decode semantic content (see Fig. 5, Table 2).

#### 3.2.2 Perceptual classifier

The classifier that was trained on the distinction between spoken and written non-words and tested on the distinction between these presentation modalities for words, was also run in three separate masks (ATL; primary auditory cortex and primary visual cortex). All results reported are above chance levels (50%, cluster corrected *p*<.05). Within the ATL, anterior portions of STG, extending into temporal pole, were able to decode between presentation formats (see Fig. 3; Table 2). The classifier results for the primary auditory cortex mask revealed an extensive cluster of voxels that could classify perceptual information in Heschl's Gyrus, planum temporale and superior temporal gyrus (see Fig. 4; Table 2). The classifier results for the primary visual cortex mask revealed an extensive cluster of voxels in occipital pole (see Fig. 5; Table 2).

To explicitly determine whether the aITG and aSTG were differentially able to classify the modality of presentation and the meaning of the stimulus, we conducted a 2×2 repeated-measures ANOVA in which we compared the prediction accuracies for each classifier output for each significant cluster. This revealed three significant effects. First, a main effect for classifier type (presentation format vs. semantic classifier; F(1,18)=36.76, *p*<.001). Second, a significant main effect of region (aSTG vs. aITG; F(1,18)=79.71, *p*<.001). Critically, we also found a significant interaction between classifier type and ATL region (F(1,18)=1087.51, *p*<.001). Post-hoc tests revealed a significant difference between aSTG and aITG for the presentation format classifier, with aSTG performing significantly better than aITG (*t*(18)=29.04, *p*<.001). There was also a significant difference between aITG and aSTG for the semantic feature classifier, with aITG performing significantly better than aSTG (*t*(18)=28.30, *p*<.001). Collectively, these analyses show a dissociation between ATL regions: aSTG classification accuracy was higher for presentation modality than word meaning, while the reverse pattern was obtained for aITG.

In addition to our ROI-based MVPA results, a whole-brain searchlight analysis was computed for both the semantic feature classifier and perceptual classifier, using the same analysis pipeline outlined for our ROI analysis. Results from the whole-brain searchlight reveal similar clusters across primary auditory cortex, primary visual cortex and anterior temporal lobe. In addition, the whole-brain analysis revealed clusters in occipital-parietal cortex and clusters extending along the temporal lobe. The unthresholded maps from the whole-brain searchlight analysis have been uploaded to the neurovault database and can be found here http://neurovault.org/collections/1970/.”

### 3.3 Univariate analysis

**Fig. 6:**
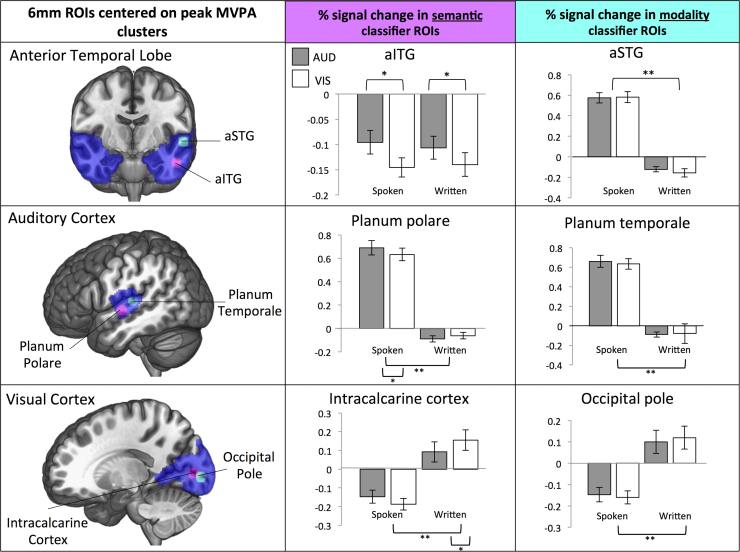
The first column shows 6 mm ROIs centered on the peak MVPA results from the searchlight analyses (shown in Fig. 3, Fig. 4, Fig. 5) for semantic classifier in magenta and modality classifier in cyan, for each of our three masks (anterior temporal lobe, primary auditory cortex and primary visual cortex) projected in blue. The centre for these ROIs are as follows; aITG seed [-50 -10 -26], aSTG seed [-58 -10 -2], planum polare [-48 -12 -4], planum temporale [-58 -24 8], intracalcarine cortex [-16 -84 4] and occipital pole [-16 -92 0]. The second column shows the univariate percent signal change for each of our four conditions within the semantic (magenta) ROI. The third column shows the univariate percent signal change for each of our four conditions with the modality (cyan) ROI. Grey bars show the results for auditory-feature words (e.g., ‘loud’) and white bars show the results for visual-feature words (e.g., ‘bright). * indicates a significant difference between auditory-features and visual-features within a modality (i.e., spoken auditory-features and spoken visual-features; *p*<.05). ** indicates a significant difference between spoken and written presentation format (*p*<.001). The unthresholded univariate maps for each condition have been uploaded to the Neurovault database and can be found here http://neurovault.org/collections/1970/. (For interpretation of the references to color in this figure legend, the reader is referred to the web version of this article.).

The searchlight results revealed that in ATL, primary auditory cortex and visual cortex, distinct regions were able to decode semantic feature type and presentation modality. As an additional complementary analysis, the percentage signal change was extracted for each condition from the pairs of clusters that were able to decode semantic feature type and modality of presentation in ATL, visual cortex and auditory cortex (generating six analyses; see Fig. 6). A 6mm sphere was centered at the peak MVPA accuracy in each of these sites (see Table 2). The ventral ATL region (encompassing aITG and aMTG, decoding feature type) showed deactivation across all four conditions, and the degree of deactivation was sensitive to meaning (auditory > visual features) but not input modality (spoken=written words). In contrast, aSTG (which decoded presentation modality) was sensitive to modality (spoken>written) but not meaning (auditory=visual features). Thus, univariate analyses also revealed a functional dissociation within ATL. We also examined regions that could decode modality of presentation and semantic feature type within primary auditory cortex (planum temporale and planum polare respectively) and primary visual cortex (occipital pole and intracalcarine cortex). All four sites showed strong effects of input modality in univariate analyses across both feature types. In addition, the intracalcarine cortex showed greater activity to words that denoted a visual property (e.g., bright) whereas planum polare showed greater activation to words that denoted an auditory property (e.g., loud). This effect of meaning in primary visual and auditory areas was only seen when the words were presented in the complementary input modality: primary visual cortex responded more to visual features when written words were presented, while primary auditory cortex responded more to auditory features when spoken words were presented. Thus, aITG was unique in showing a pattern across both multivariate and univariate analyses consistent with the predictions for an amodal ‘hub’: i.e., sensitivity to meaning and insensitivity to presentation modality.

### 3.4 Resting state fMRI

**Fig. 7:**
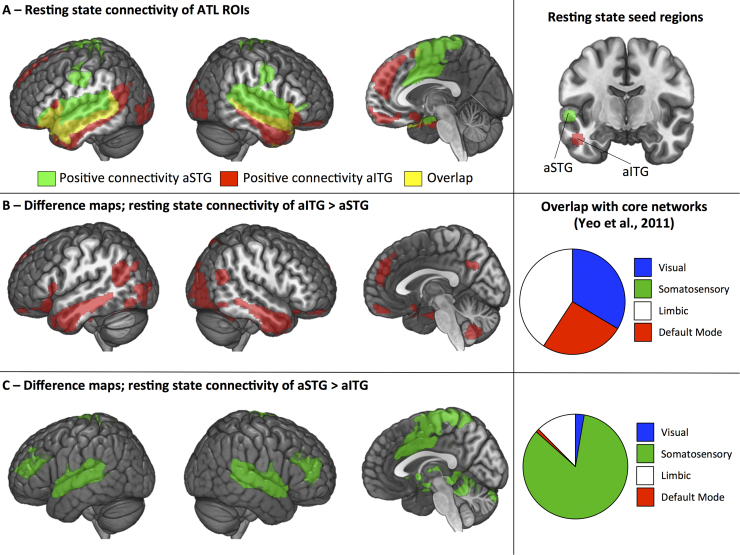
Resting state connectivity maps projected on rendered brain, displaying (from left-to-right) left hemisphere, right hemisphere, medial view. Maps thresholded at z=2.3, cluster corrected p<.01. (A) Resting state connectivity from two ATL regions connectivity maps; green seed=aSTG (taken from peak accuracy for modality classifier within anterior temporal lobe) and red seed=aITG (taken from peak accuracy for semantic classifier within anterior temporal lobe) - the seed locations are highlighted on the right. (B) Subtraction analysis from two ATL connectivity maps; red=aITG>aSTG. Pie chart on the right shows proportion of overlapping voxels for this difference map with core networks taken from Yeo et al. (2011). These four networks include two sensory maps (Visual, Somatosensory), Limbic and Default Mode Network. (C) Subtraction analysis from two ATL connectivity maps; green=aSTG>aITG. Pie chart on the right shows proportion of overlapping voxels for this difference map with core networks taken from Yeo et al. (2011). (For interpretation of the references to color in this figure legend, the reader is referred to the web version of this article.).

To provide a better understanding of the neural architecture that supported the functional distinction between aSTG (effect of input modality) and aITG (effect of semantic feature type), we explored the connectivity of these regions in resting state fMRI (see Fig. 7) by placing spherical ROIs at peaks in the MVPA analysis. The aSTG seed showed significant positive connectivity across the entire length of STG through primary auditory cortex and into supramarginal gyrus (SMG). It coupled with posterior and anterior regions of MTG, pre- and postcentral gyrus, supplementary motor cortex and anterior cingulate gyrus and deactivation with visual regions, including lateral occipital cortex, intracalcarine cortex, occipital fusiform gyrus (OFG) and temporal occipital fusiform gyrus, as well as posterior cingulate and precuneous. In contrast, the aITG site showed connectivity with core parts of the default mode network and multimodal semantic regions, including angular gyrus, posterior parts of MTG and ITG, temporal pole extending medially to include hippocampus and anterior parahippocampal gyrus, and anterior and inferior prefrontal regions, including orbital cortex and left inferior frontal gyrus (LIFG). This seed also coupled with lateral visual regions (e.g., LOC and occipital fusiform gyrus). Table 3 presents location and size of each of these clusters.

To investigate the differences between these two ATL maps a difference analysis was performed (Fig. 7B). The contrast of aSTG > aITG identified bilateral superior temporal and frontal polar regions. The contrast aITG > aSTG revealed bilateral inferior and middle portions of the temporal lobe and multimodal semantic sites including angular gyrus, pMTG and LIFG. These differences resemble resting state differences for aSTG and vATL reported by Jackson et al. (2016), helping to validate the functional dissociation we observed using MVPA.

To further interrogate the assumption that aITG exhibits a connectivity profile consistent with an amodal region, whereas aSTG is connected to sensory regions, we looked at the similarity between our two ATL difference maps (see Fig. 7B and C) and that of four core networks taken from Yeo. et al. (2014). These included two networks sensitive to sensory input (visual, somatosensory) and two networks thought to be crucial in the generation of cognitive states that do not rely on sensory inputs for their mental content (limbic and default mode network) (for a review see Andrews-Hanna et al. (2014)). The results, outlined in Figs. 7B and 7C, indicated substantial overlap between the sensory networks (namely somatosensory) and aSTG. In contrast, aITG showed substantial overlap with limbic and DMN networks.

## 4 Discussion

The current study used multiple imaging methods to identify regions in the anterior temporal lobe (ATL) and primary sensory regions that showed the pattern expected for the semantic hub of the hub and spokes model (Patterson et al., 2007). In an fMRI study, participants listened to or viewed words that referred to either visual or auditory features (e.g., bright or loud). Multivoxel pattern analysis (MVPA) revealed a dissociation between (i) anterior inferior temporal gyrus (aITG), which could classify semantic categories relating to feature type (e.g., auditory features like “loud” as being different from visual features like “bright”) across auditory and visual inputs and (ii) anterior superior temporal gyrus (aSTG), which was sensitive to input modality across meaningful and meaningless items. This dissociation within ATL was further supported by univariate contrasts and patterns of resting state connectivity: aSTG showed a stronger response to spoken than written inputs and was functionally coupled to an auditory-motor network (somatosensory network; Yeo et al., 2014), while aITG was insensitive to input modality and showed substantial connectivity with regions in the default mode network and limbic network, plus some overlap with visual regions (see Jackson et al. (2016), for similar findings).

Our findings make an important contribution to our understanding of the neural basis of semantic cognition in three ways: (1) We provide evidence that conceptual knowledge, extracted from different modalities of input across many learning experiences, is represented within *ventral* portions of ATL which act as a ‘hub’ (Patterson et al., 2007, Rogers et al., 2004). (2) Across converging methods, we observe a functional dissociation between ventral and superior portions of ATL and provide evidence that these regions are situated within distinct large-scale cortical networks. (3) Responses in primary visual and auditory cortex confirm the contribution of these ‘spoke’ regions to semantic processing.

According to the hub and spoke model (Patterson et al., 2007), conceptual knowledge depends on the co-activation of spoke regions that convey information about specific unimodal and multimodal features of concepts, and an ATL hub which integrates these features to form amodal conceptual representations that are independent of specific sensory input. Studies of patients with semantic dementia (SD) provided the original motivation for this proposal yet neuropsychological methods are not especially well-suited to the precise localization of amodal conceptual representations given the widespread atrophy in this condition. Nevertheless, the degree of semantic impairment correlates with hypometabolism in ventral rather than superior portions of ATL across patients (Mion et al., 2010), suggesting that ventral ATL could be the critical substrate for amodal knowledge. Relevant evidence is also provided by univariate fMRI analyses of the ATL response to verbal comprehension tasks in healthy participants, which show multiple peak responses in both ventral ATL and aSTG, often to the same contrasts (Binney et al., 2010, Hoffman et al., 2015; Visser and Ralph, 2011). Semantic matching and naming tasks have also shown multiple peak responses in the ATL with the more superior ATL region being involved in object naming and the more ventral region in semantic matching (Sanjuán et al., 2015). Furthermore, the differential patterns of functional connectivity across ATL regions have been observed by both Jackson et al., (2016) and Pascual et al., (2015).

Our findings therefore add to existing knowledge by showing a dissociable response in these two regions: only the ventral ATL site showed a pattern consistent with the representation of conceptual information, since it was able to classify responses according to semantic category (i.e., feature type, not input modality). In univariate analyses, this aITG site also showed deactivation (arguably due to the use of rest rather than an active baseline; Visser et al., 2010; Humphreys et al., 2015) for both auditory and visual feature types, irrespective of whether these words were spoken or written – and the magnitude of this deactivation was greater for visual than auditory features. Finally, this site showed stronger functional connectivity at rest with the default mode and limbic systems, as expected for a region implicated in amodal conceptual processing. Therefore, our combination of functional and resting state methods provides novel converging evidence that anterior ventral temporal areas allow different sensory representations to be integrated to form ‘amodal’ conceptual representations (particularly for auditory features, see limitations below).

Previous studies have used MVPA to explore the neural basis of semantic processing, and have identified a conceptual response in ATL using classification of stimuli within a single presentation modality (Coutanche and Thompson-Schill, 2014, Peelen and Caramazza, 2012). Other studies, examining semantic cognition across modalities of presentation (Devereux et al., 2013, Fairhall and Caramazza, 2013, Man et al., 2015), have largely not observed effects in ATL. An exception is a recent crossmodal MVPA study, investigating Dutch-English bilinguals (Correia et al., 2014). The research tested whether patterns of activity related to the distinction between spoken nouns in one language (e.g., “horse” vs. “duck” in English) could accurately predict the same distinction in the other language (e.g., “paard” vs. “eend” in Dutch). Consistent with our findings, the cross-language classifier revealed a significant cluster in the left ATL. This largely fell within mid-superior temporal pole rather than the more ventral region we identified in our analysis, perhaps because aSTG is an important interface between semantic processing and other aspects of language.

**Fig. 8:**
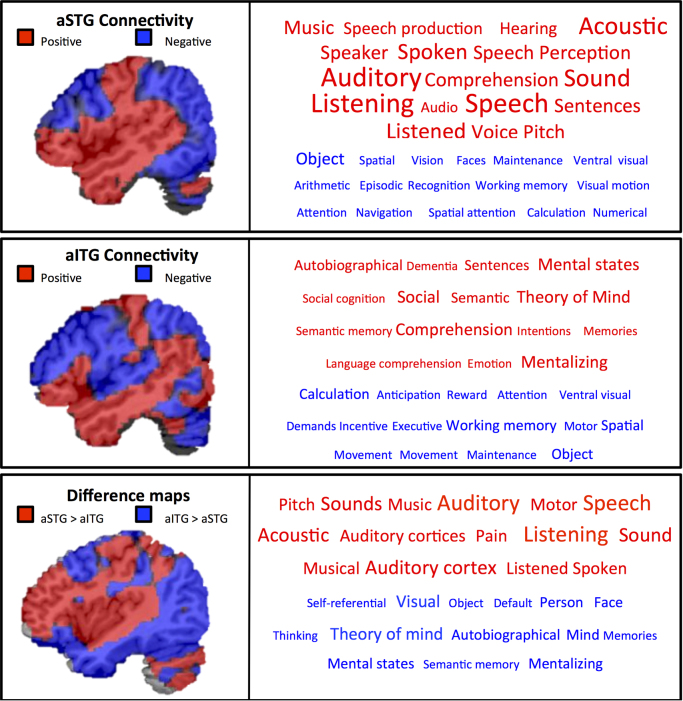
Decoding the functions of two ATL components (aSTG and aITG) using automated fMRI meta-analyses (NeuroSynth, Yarkoni et al., 2011). This software computed the spatial correlation between each ATL component unthresholded zstat mask (shown on the left; red = positive correlation and blue=negative correlation) and every other meta-analytic map (*n*=11406) for each term/concept stored in the database (e.g., semantic, language, memory and sensory). The 15 meta-analytic maps exhibiting the highest positive correlation (red words) and negative correlation (blue words) for each sub-system mask were extracted, and the term corresponding to each of these meta-analyses is shown in the respective box (shown on the right). The font size reflects the size of the correlation (ranging from *r*=0.10 to 0.45 for positive correlations (red) and *r*=−0.05 to −0.2 for negative correlations (blue), in increments of 0.05). This allows us to quantify the most likely reverse inferences that would be drawn from these functional maps by the larger neuroimaging community. (For interpretation of the references to color in this figure legend, the reader is referred to the web version of this article.).

Analyses of resting state connectivity from the ATL regions that were able to classify input modality (aSTG) and semantic feature type (aITG) revealed that these two sites lie within distinct large-scale functional networks. A similar dissociation between the resting state connectivity of ventral ATL and anterior STG was recently reported by Jackson et al., (2016), providing further evidence for the validity of the functional dissociation in ATL that we observed using MVPA. To quantify the interpretation of the functional connectivity of the aSTG and aITG connectivity maps, we performed a decoding analysis using automated fMRI meta-analytic software NeuroSynth (see Fig. 8). Meta-analytic decoding of these spatial maps revealed that our aSTG connectivity map correlated with terms related to language (e.g., sentence, comprehension) and auditory processing (e.g., speech, sound) whilst anti-correlating with other modality information (e.g., visual, spatial) and memory (e.g., working memory, episodic). In contrast, the aITG connectivity map correlated with terms related to memory (e.g., semantic, autobiographical) and social processes (e.g., theory of mind, social cognition) terms, whilst anti-correlating with modality-specific (e.g., ventral visual, motor, spatial) and executive terms (e.g., maintenance, demands). This is consistent with previous findings that relate aSTG to speech comprehension, language and sensory processing (Patterson and Lambon Ralph, 1999, Jobard et al., 2007, Scott and Johnsrude, 2003, Scott et al., 2003, Scott et al., 2000, Spitsyna et al., 2006) and aITG to semantic processing but not sensory experience (Patterson et al., 2007, Visser et al., 2010). Furthermore, the differences in function across temporal areas as revealed by the Neurosynth database seem to align with differences in the white-matter terminations (see Bajada et al., 2016). These findings confirmed associations between (i) the network anchored in the aSTG and auditory processing and speech perception, plus (ii) the aITG network and more abstract domains (such as social cognition, theory of mind, or mental states).

Thus, the putative semantic ‘hub’ in ventral ATL was functionally coupled to aspects of cortex that specialize in forms of stimulus-independent higher order cognition, including angular gyrus (AG) and posterior and anterior areas on the medial surface that correspond to the midline core of the so-called default mode network (DMN)(see also Hurley et al., 2015). This network is known to be deactivated by input (Raichle et al., 2001) and is thought to be crucial in the generation of cognitive states that do not rely on sensory information for their mental content (for a review see Andrews-Hanna et al., 2014). Tasks which are associated with the default mode network include those that depend on episodic memory, semantic processing, mental state attribution as well as states of spontaneous thought studied under the rubric of mind-wandering / daydreaming (Spreng et al., 2009, Raichle, 2015). Although previous literature has shown that connectivity to the AG may not be due to shared semantic processing (Humphreys et al., 2015). Therefore, as many cognitive states that involve the DMN are stimulus-independent in nature, their association with ventral ATL both in terms of functional connectivity and their meta-analytic decoding is consistent with the view that this region supports semantic processing across different input modalities and may form conceptual representations that are not tied to a specific input modality (see Margulies et al., 2016). In contrast, aSTG showed greater functional connectivity with auditory and motor regions and this spatial map was associated with auditory processing and language tasks, as opposed to amodal tasks, in the meta-analytic decoding. Therefore, our combination of functional and resting state methods provides novel converging evidence that anterior ventral temporal areas allow different sensory representations to be integrated to form ‘amodal’ conceptual representations.

As discussed, the hub and spoke model (Lambon Ralph et al., 2010, Patterson et al., 2007, Rogers et al., 2004) makes novel predictions about the contribution of the ATL to amodal conceptual knowledge, but it also anticipates an important role for modality-specific ‘spoke’ regions in visual and auditory cortex, in line with many influential accounts of semantic processing (Damasio, 1989, Martin, 2007, Meteyard et al., 2012, Pulvermüller, 2013). Furthermore, the involvement of both hub and spoke regions in semantic representations has been shown using TMS (Pobric et al., 2010). In line with this view, MVPA revealed regions that responded to meaning in both ventral parts of ATL (putative ‘hub’) and in primary visual and auditory regions (putative ‘spokes’). In addition, even though the putative ‘spoke’ regions (i.e., voxels sensitive to meaning) were adjacent to areas that coded for input modality, the specific voxels that could classify meaning and input modality were largely different. These findings do not readily support traditional ‘strong’ embodied accounts that equate semantic representations with traces of perceptual/motor experience (for a review, see Meteyard et al. (2012)) since this would suggest a greater degree of overlap between the results of these two classifiers. While our data suggests that sensory systems appear to play a critical role in the representation of meaning, they also suggest that perceptual experience and imagery generated as part of semantic retrieval may be distinguishable on the basis of differences in the patterns of activity in sensory cortex.

One potential limitation of our study is that we did not observe evidence that aITG responds to both auditory and visual semantic features in the univariate contrasts: this site showed deactivation for both feature types that was greater for visual features. Thus, the strongest evidence for the aITG as an amodal hub is provided by the MVPA results and our meta-analytic decoding of this region's pattern of distinct functional connectivity, and not the univariate analyses. Our design was optimized for decoding rather than univariate effects – as we focused on obtaining the maximum number of blocks for MVPA and did not employ a high-level non-semantic baseline which would have allowed us to recover semantic activation in ATL for both auditory and visual features from a contrast (Humphreys et al., 2015). Since we found that aITG responds more to auditory features (words such as “loud”) than visual features (words such as “bright”), it remains unclear whether aITG reflects the meanings of auditory features alone, or both feature types equally. Future studies might allow these possibilities to be disentangled using a high-level baseline with which both feature types can be compared (e.g. Jackson et al., 2015).

## 5 Conclusion

Collectively, our findings from both pattern classification and resting-state connectivity provide converging evidence that sub-regions of the ATL support different aspects of semantic processing. Anterior ITG and MTG capture meaning independent of input modality, consistent with the fact that semantic dementia patients (who have multimodal semantic impairment) have considerable atrophy in this same region of ATL (Binney et al., 2010, Galton et al., 2001). In contrast, aSTG exhibited a degree of modality specificity: this structure, which is known to be important for understanding speech and environmental sounds, does not fulfil the criteria for an amodal semantic hub. Finally, the current results provide evidence for modality-specific spokes regions within the vicinity of primary auditory and visual cortex (intracalcarine cortex and planum polare respectively). However, the specific voxels that could classify between each condition (presentation format and semantic feature) were largely different. These findings challenge traditional embodied accounts (Pulvermuller, 2005) that attempt to equate semantic representations with traces of perceptual/motor experience, and instead support the view that the richness of semantic cognition arises at least in part from abstraction away from specific input modalities in ventral regions of the anterior temporal lobe.

## Funding

The research was supported by BBSRC grant BB/J006963/1. Jefferies was supported by a grant from the European Research Council (SEMBIND - 283530). The publication was part-funded by a grant from the John Templeton Foundation, “Prospective Psychology Stage 2: A Research Competition” to Martin Seligman. The opinions expressed in this publication are those of the author(s) and do not necessarily reflect the views of the John Templeton Foundation. The authors declare no competing financial interests.

## Figures and Tables

**Table 1:** Mean psycholinguistic properties of stimuli (SD in parentheses).

Property		**Auditory feature words**		**Visual feature words**	**Non-words**
Example		“loud”		“shiny”	“brodic”
Log frequency		2.27 (1.05)		2.54 (.82)	N/A
Length		5.25 (.76)		5.50 (.80)	5.88 (1.17)
Syllables		1.88 (.45)		1.63 (.49)	2.00 (.50)
Age of acquisition		7.17 (2.70)		6.85 (2.76)	N/A
Familiarity		4.43 (.63)		4.40 (.51)	N/A
Emotional Valence		3.18 (.70)		3.3 (.67)	N/A
Levehnstein distance		5.11 (.94)		6.00 (1.25)	5.89 (.86)
Behavioural feature-rating (auditory)		4.45 (.61)*		1.15 (.04)*	N/A
Behavioural feature-rating (visual)		1.65 (.32)*		4.77 (.19)*	N/A
Behavioural feature-rating (haptic)		1.5 (.39)		1.76 (.72)	N/A
Behavioural feature-rating (taste)		1.19 (.07)		1.21 (.09)	N/A

Footnote: Log frequency=log-transformed lemma frequencies from the SUBTLEX database (Brysbaert, New and Keuleers, 2012;

http://expsy.ugent.be/subtlexus). Length=number of letters. Age of acquisition (AoA norms; Kuperman et al., 2012). Part of

speech also taken from SUBTLEX database. Familiarity, emotional valence and behavioural feature rating (auditory; visual;

haptic; taste) were obtained from a behavioural experiment with a separate cohort of participants from the fMRI study.

These were scored on a Likert-scale (1–5).

* Wilcoxon signed rank tests revealed a significant difference between auditory-feature and visual-feature conditions (*p*<.001).

**Table 2:** Centre Voxel Coordinates of Highest Decoding Sphere in the Searchlight Analyses.

**Condition**	**Mask**	**Cluster Peak**	**Extended Cluster Regions**	**Cluster Extent**	**Z-score**	**Acc (%)**	**x**	**y**	**z**
Semantic Feature									
	ATL	L Anterior ITG/MTG	L Heschls gyrus, L putamen	478	4.91	61.22	-50	-10	-26
	ATL	R Temporal pole	R Anterior parahippocampal gyrus, R Anterior MTG, R Anterior STG.	416	4.58	61.05	42	12	-24
	Auditory	L Planum polare	L Heschls gyrus, L Planum temporale	88	3.92	59.53	-48	-12	-4
	Visual	L Intracalcarine cortex	L Lingual gyrus	81	4.26	61.18	-18	-84	4
									
Presentation format									
	Visual	L Occipital pole	L Occipital fusiform gyrus, L Inferior lateral occipital cortex.	607	4.3	58.57	-16	-92	0
	Auditory	L Planum temporale	L Heschl's gyrus, R Planum Temporale, R Heschl's gyrus,	581	4.97	59.85	-58	-24	8
	ATL	L Anterior STG	L Temporal pole, R Anterior STG	66	2.8	58.36	-58	-10	-2

Footnote: Highest decoding accuracy clusters for semantic feature (AUD vs. VIS) and presentation format (spoken vs. written words) analysed separately. Semantic feature classifier was trained on the distinction between spoken AUD vs. spoken VIS and tested on written AUD vs. written VIS (and vice versa). Presentation format classifier was trained on the distinction between written non-words vs. spoken non-words and tested on spoken words vs. written words. Results are thresholded at p<.05 (cluster corrected). L=left, R=right. As well as peak accuracy (reported under the ‘Cluster Peak’ column), the ‘Extended Cluster Regions’ includes all significant regions within each ROI. In addition to the searchlight analyses reported in the table, a further searchlight analysis was run on the distinction between all spoken vs. all written items. This revealed accuracies as high as 99.6% in primary sensory regions and 93.2% in ATL. The unthresholded MVPA maps for each searchlight have been uploaded to the Neurovault database and can be found here http://neurovault.org/collections/1970/.

**Table 3:** Coordinates of peak clusters in the resting-state connectivity analyses.

**Seed Region**	**Cluster**	**Cluster Extent**	**Z-score**	**x**	**y**	**z**
aSTG	*Increased Correlation*					
	L. aSTG	15745	12.3	-54	2	-10
	R. Temporal pole	12970	9.24	52	8	-14
	Cingulate Gyrus	7618	7.02	-4	12	32
	*Reduced Correlation*					
	L. Cuneal cortex	26667	6.19	-20	-74	32
	R. Superior frontal gyrus	4128	4.69	20	12	52
	L. Middle frontal gyrus	2259	4.53	-32	10	50
	L. Lateral occipital cortex, inferior	1457	5.46	-46	-70	-12
						
aITG	*Increased Correlation*					
	L. aITG/MTG	20324	13.1	-50	-10	-26
	L. Frontal pole	2899	7.22	-10	50	32
	L. Occipital fusiform gyrus	1981	4.49	-26	-82	-8
	*Reduced Correlation*					
	Postcentral gyrus	3725	4.44	0	-54	74
	R. Frontal pole	2717	5.07	42	54	12
	L. IFG, pars triangularis	2118	5.17	-46	35	16
	R. Cingulate gyrus	1276	4.44	12	32	16
	L. Angular gyrus	783	4.39	-40	-50	42
	L. Superior parietal lobule	769	3.94	-30	-48	-56
	L. Middle frontal gyrus	724	4.72	-28	8	60
	R. Middle frontal gyrus	626	4.16	30	12	56

Footnote: The table shows peak clusters in the resting-state connectivity analysis from two seed regions; aSTG and aITG. Results are thresholded at p<.01 (cluster corrected).

L=left, R=right.
